# New Strategy for the Covalent Immobilisation of Phenolic Compounds on Silica Particles to Fight Against Foodborne Pathogens

**DOI:** 10.3390/foods14010045

**Published:** 2024-12-27

**Authors:** Alejandro Rivas, Héctor Gómez-Llorente, Oumaima Moumane, Jose Manuel Barat, Édgar Pérez-Esteve

**Affiliations:** Instituto Universitario de Ingeniería de Alimentos—Food UPV, Universitat Politècnica de València, Camino de Vera s/n, 46022 Valencia, Spain; alriso@upv.es (A.R.); hecgollo@upv.es (H.G.-L.); oumou@etsiamn.upv.es (O.M.); edpees@upv.es (É.P.-E.)

**Keywords:** thymol, carvacrol, covalent immobilisation, food preservatives, foodborne pathogens, apple juice

## Abstract

The immobilisation of essential oil components (EOCs) on food-grade supports is a promising strategy for preserving liquid foods without the drawbacks of direct EOC addition such as poor solubility, high volatility, and sensory alterations. This study presents a novel method for covalently immobilising EOCs, specifically thymol and carvacrol, on SiO_2_ particles (5–15 µm) using the Mannich reaction. This approach simplifies conventional covalent immobilisation techniques by reducing the steps and reagents while maintaining antimicrobial efficacy and preventing compound migration. The antimicrobial effectiveness of the EOC–SiO_2_ system, applied as an additive, was tested against foodborne pathogens (*Escherichia coli*, *Salmonella enterica*, *Staphylococcus aureus,* and *Listeria monocytogenes*) inoculated into phosphate buffer solution and fresh apple juice. The results showed high antimicrobial activity, with inactivation exceeding 4-log reductions, depending on the EOC type, target microorganism, and medium. Moreover, the addition of functionalised particles did not affect the juice organoleptic properties. This study demonstrates that the Mannich reaction is an effective method for developing antimicrobial systems based on the covalent immobilisation of EOCs on silica particles, and offers a practical solution for food preservation without compromising food quality.

## 1. Introduction

In recent years, foodborne outbreaks involving pathogens like *Escherichia coli*, *Listeria monocytogenes*, *Salmonella enterica*, and *Staphylococcus aureus* have increased, driven by factors like globalised food supply chains, poor food safety practices, rising demand for minimally processed foods, and environmental changes that support pathogen survival. Notably, outbreaks linked with apple juice have raised concerns despite its acidic and stable nature [1,2]. Therefore, controlling these pathogens is crucial for ensuring both product quality and microbiological safety in the food industry, especially in the apple juice sector.

Traditional preservation methods primarily rely on heat treatments (e.g., pasteurisation and sterilisation) or the addition of synthetic preservatives. Heat treatments effectively reduce microbial load and deactivate enzymes to stabilise food, but can also lead to a loss of nutrients, such as vitamins [3], and negatively affect the taste and flavour [4]. The carbon footprint of preservation techniques is another important consideration [5]. Finally, the shift from synthetic to natural preservatives in the food industry is driven by growing concerns about health, consumer demand for cleaner labels, environmental sustainability, and regulatory changes [6].

In this context, employing essential oil compounds (EOCs), which are a group of substances containing terpenes, terpenoids, phenylpropane-derived aromatic compounds, and aliphatic compounds, is seen as a promising alternative. Their wide range of biological activities including bactericidal, virucidal, fungicidal, antiparasitic, insecticidal, and antioxidative effects makes them a potential solution to overcome the limitations of traditional food preservation methods [7,8]. However, despite the proven effectiveness of these antimicrobials in culture media, their direct incorporation into food products presents certain challenges such as their poor solubility, their high volatility, and their ability to modify sensory food attributes [9,10]. One of the strategies to improve their applicability in foods is the covalent immobilisation of these compounds on food-grade supports. Immobilisation consists of anchoring the active compound onto the surface of supports through a covalent bond to prevent it leaching into the food matrix [11]. The effectiveness of immobilised antimicrobial systems as food preservatives and processing aids to control or prevent microbial spoilage in food and water is well-established. These systems have a demonstrated ability to suppress both fungi (*Z. bailii* and *Z. rouxii*) and pathogenic microorganisms like *E. coli* in apple juice [12,13,14]. Their effectiveness in counts reduction when using immobilised particles as filtering aids in apple juice [15,16], white wine [17], craft beer [12], or drinking water [18] has also been indicated.

The immobilisation procedure for EOCs involves forming imine bonds between the aldehyde group of the EOC and a silane-coupling agent, which bonds the EOC to the support surface. EOCs with aldehyde groups, like vanillin and cinnamaldehyde, can be covalently immobilised through chemical interfacial reactions. However, EOCs without suitable chemical groups, such as eugenol, carvacrol and thymol, must be modified by adding an aldehyde moiety [12]. By way of example, eugenol is modified using the Reimer–Tiemann reaction, while carvacrol and thymol undergo direct formylation with paraformaldehyde [12]. To enhance stability, imine bonds are reduced to amine bonds with sodium borohydride. This immobilisation process involves many steps and a significant number of chemical reagents.

Alternatively, the Mannich reaction, which has been successfully used to develop new antibacterial and antifungal drug candidates from phenolic EOCs, could offer a more efficient derivatisation route [19,20]. This reaction requires a suitable nucleophile, a reactive aldehyde, and a primary or secondary amine [21]. The electron-rich aromatic ring of phenols facilitates the Mannich reaction in ortho- or para-positions by leading to effective derived molecules [22]. Notably, the Mannich reaction has not been previously applied for the covalent immobilisation of EOCs on silica supports, and such action could reduce the number of stages in the synthesis procedure, the number of chemical reagents, and therefore, could also lower the production costs and environmental impact.

The objective of this study was to evaluate the efficiency of the Mannich reaction for the rapid and effective functionalisation of silica particles with carvacrol and thymol. It also aimed to assess the antimicrobial activity of the resulting particles against common foodborne pathogens including *Escherichia coli*, *Salmonella enterica*, *Staphylococcus aureus*, and *Listeria monocytogenes* in fresh apple juice.

## 2. Materials and Methods

### 2.1. Materials

(3-Aminopropyl) triethoxysilane (APTES), 2,4-dinitrophenylhydrazine (DNPH), dimethyl sulfoxide (DMSO), resazurin sodium salt, sodium borohydride, carvacrol, and thymol were provided by Sigma-Aldrich (Madrid, Spain). Acetonitrile, formaldehyde 37% (*v*/*v*), methanol, silica oxide (5–15 µm), and microbiological media grade (cation-adjusted Mueller–Hinton broth (CAMHB), tryptone soya agar (TSA), tryptone soya broth (TSB), plate count agar (PCA), yeast extract (YE), and phosphate-buffered saline 1× (PBS) were provided by Scharlab (Barcelona, Spain).

### 2.2. Synthesis of the Silica Microparticles Functionalised with Antimicrobial Compounds

In this study, three EOCs were utilised: carvacrol, thymol (major components of *Lamiaceae* family plants), and eugenol (a major component of clove and cinnamon oil). These compounds were selected for their well-documented antimicrobial properties and their status as “generally recognised as safe” (GRAS) components.

**Figure 1:**
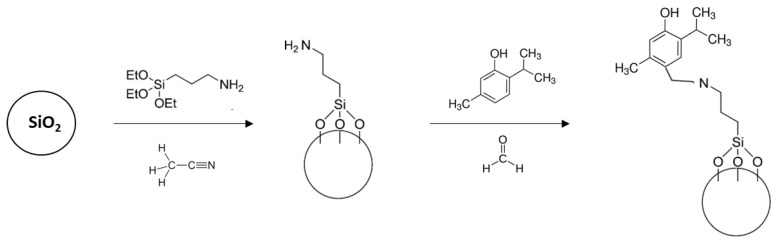
Synthesis of the silica microparticles functionalised with thymol.

A two-step synthetic procedure was followed to prepare antimicrobial devices (Figure 1). In the first step, APTES was anchored onto the surface of the silica oxide particle. In a second step, carvacrol and thymol were reacted with APTES anchored onto the support through the Mannich reaction.

#### 2.2.1. Surface Silanisation Synthesis

During a typical synthesis, 1 g of silica particles (5–15 µm) was suspended in 25 mL of acetonitrile in a round-bottomed flask. Then, 8.54 mmol of APTES was added and the final mixture was stirred for 2 h at room temperature. Finally, solids were centrifuged (9500 rpm for 5 min) and washed with water until a neutral pH and with acetonitrile. Finally, the solid was dried at room temperature in a vacuum overnight to yield SiO_2_-APTES.

#### 2.2.2. Synthesis of the EOC-Functionalised Silica Particles

The immobilisation of EOCs on the surface of **SiO_2_-APTES** was carried out by the Mannich reaction. A methanolic solution of EOC (carvacrol or thymol), formaldehyde (37%), and **SiO_2_-APTES** was mixed properly and refluxed for 24 h at 60 °C. Microparticles were washed with acid distilled water (pH 4) until the non-leaching of EOCs and formaldehyde components. Finally, the solid was dried at room temperature in a vacuum overnight to yield **SiO_2_-EOC**.

#### 2.2.3. Optimisation of the EOC Immobilisation Process on Silica Particles

In order to minimise formaldehyde use while retaining the particles’ antimicrobial activity, an optimisation of the synthesis procedure of the **SiO_2_-EOC** particles was carried out. To achieve this, particles were synthesised with varying ratios of EOC, formaldehyde (37%), and **SiO_2_-APTES**, namely 1:1:1, 1:0.75:1, 1:0.50:1, 1:0.25:1, and 1:0.10:1 to yield reactions A, B, C, D, and E, respectively.

Three different parameters were then measured for use as the selection criteria: (i) the number of washes required to eliminate detectable EOCs and formaldehyde in washing water; (ii) the degree of functionalisation; and (iii) the particles’ antimicrobial activity against *E. coli* was determined according to Section 2.4.2.

Each washing cycle consisted of adding 30 mL of acid distilled water to a Falcon tube containing one gram of particle, vortexing, and centrifuging (9500 rpm for 5 min). Then, the concentrations of EOCs and formaldehyde were analysed by high-performance liquid chromatography (HPLC; see Section 2.2.5).

#### 2.2.4. Silica Microparticle Characterisation

Bare and functionalised silica supports were analysed using three standard methods: morphological examination, zeta potential (ζ) measurement, and functionalisation quantification through elemental analysis. Morphological observations were conducted via field emission scanning electron microscopy (FESEM), with images captured using a Zeiss Ultra 55 microscope (Carl Zeiss NTS GmbH, Oberkochen, Germany) operating in secondary electron mode.

The ζ of both the bare and functionalised mesoporous silica particles was measured using a Zetasizer Nano ZS (Malvern Instruments, Malvern, UK). For these measurements, samples were prepared by dispersing the particles in distilled water at a concentration of 1 mg/mL and sonicated for 2 min to prevent aggregation. Zeta potential values were derived from particle mobility using the Smoluchowski model [13], with measurements conducted at 25 °C and repeated three times.

The extent of functionalisation of the silica particles, expressed as mg of EOC per gram of SiO_2_, was calculated from the results of the elemental analysis (C, H, and N) performed with a Vario EL III Element Analyzer (Elementar Analysensysteme GmbH, Langenselbold, Germany).

#### 2.2.5. Leaching of Immobilised Antimicrobials

The possible leaching of both immobilised bioactive compounds (carvacrol or thymol) and formaldehyde was determined by HPLC with a UV detector. The chromatographic analysis was conducted using a Hitachi LaChrom Elite HPLC system (Hitachi Ltd., Tokyo, Japan) comprising an auto-sampler (model L-2200) and a UV detector (model L-2400). A Scharlab KromaPhase 100 C18 column (150 × 4.6 mm i.d., 5 μm, 100 Å), with a C18 guard column (10 mm × 4.6 mm), was utilised. The maintained flow rate was 1.0 mL/min at 25 °C, with an injection volume of 10 μL.

For the analysis of EOC leaching, a procedure described by Xing [23] was employed with minor modifications. To identify carvacrol and thymol, an isocratic elution with 50% of A and 50% of B [where A = deionised water (Aquinity deioniser, Membrapure GmbH, Berlin, Germany) and B = methanol] was employed. The UV detection wavelengths were set at 275 nm and 277 nm for carvacrol and thymol, respectively.

For the formaldehyde analysis, derivatisation of the compound with DNPH was necessary because the compound does not present any chromophore element. The derivatisation and quantification of possible formaldehyde leaching were performed following the method described by Nageswari [24] with minor changes. In this work, an isocratic elution with 50% of A and 50% of B [where A = deionised water (Aquinity deionizer, Membrapure GmbH, Berlin, Germany) and B = acetonitrile] were applied, and UV detection was set at 360 nm.

Carvacrol, formaldehyde, and thymol were quantified by the external standard method via the calibration curve (0.5, 1, 5, 10, 25, 50, 100, 250, and 500 mg/L), which correlated the peak area with the concentration of the compound [25]. The results were expressed as carvacrol, formaldehyde, and thymol leaching (mg/mL). For each of the immobilisation reactions, HPLC analyses were performed in triplicate (n = 3).

### 2.3. Microbiological Assays

Strains *L. monocytogenes* (CECT 935), *E. coli* (CECT 5947), *S. enterica* (CECT 443), and *S. aureus* (CECT 976) were obtained from the Colección Española de Cultivos Tipo/Spanish Type Culture Collection (CECT; Valencia, Spain). The bacterial strain was reconstituted following the CECT instructions, and the bacterial stock was stored at 4 °C in PCA before being used. To obtain an inoculum with an approximate microbial density of 10^9^ CFU/mL, a colony was transferred to a tube with 10 mL of TSB to be incubated at 37 °C for 24 h. After incubation, the cell concentration of the inoculum was checked by determining the optical density at 600 nm in a Helios Zeta UV–Vis instrument (Thermo Scientific, Hampton, NH, USA).

#### 2.3.1. Determination of the MIC and MBC of Free EOCs

The determination of the minimum inhibitory concentration (MIC) of the free EOCs was carried out by the addition of resazurin dye as a redox indicator [26]. Active bacterial cells reduce non-fluorescent resazurin (blue) to fluorescent resorufin (pink), which can be further reduced to hydroresorufin [27] to provide a direct quantifiable measure of bacterial metabolic activity.

The resazurin method was carried out on sterile 96-well polystyrene round-bottomed microtiter plates using 2-fold dilutions ranging from 10 mg/mL to 0.018 mg/mL, prepared in CAMHB from an EOC stock solution (300 mg/mL) in DMSO. After 2-fold dilutions, each well contained 50 µL of the EOC/CAMHB broth. The assay included positive (diluted standardised inoculum) and negative (CAMHB) controls. Inocula were prepared following the CSLI recommendation, according to which the OD600 value was adjusted to the equivalent of 10^8^ CFU/mL, which was determined from a calibration curve for each microorganism [28]. The standardised microorganism suspension was then diluted by 1:100 in MHB broth. Then, 50 µL of the adjusted OD600 bacterial suspension was added to all the wells containing EOC as well as the control wells, which resulted in approximately 5 × 10^5^ CFU/mL. After incubation for 24 h at 37 °C, resazurin (0.015%) was added to all the wells (30 µL per well) and further incubated for 2–4 h to observe a colour change. Following incubation, the columns with no colour change (blue resazurin colour remained unchanged) were scored for being above the MIC value. The minimum biocidal concentration (MBC) was determined by directly plating the content of the wells with concentrations higher than the MIC value. The MBC value was determined when there was no colony growth from the directly plated contents of the wells.

#### 2.3.2. Determination of the Antibacterial Activity of Immobilised Antimicrobials

The antimicrobial activity of the immobilised EOCs was determined by the macrodilution method. Two levels of concentrations were tested for each EOC, MIC and MBC. Equivalent amounts of the immobilised EOCs were calculated according to the results obtained when characterising the degree of functionalisation. The particle was added to 1 mL of PBS inoculated with the foodborne pathogen (cell density of approx. 10^5^ CFU/mL) to be incubated with orbital stirring (150 rpm) at 37 °C for 24 h. All treatments were performed in triplicate and all assays included positive and negative controls.

After incubation, viable cell numbers were determined as colony-forming units (CFUs) by the spread plate technique using TSA for *E. coli* and *S. enterica* or TSA with yeast extract (0.6%) (TSAYE) for *S. aureus* and *L. monocytogenes*. Petri plates were incubated at 37 °C for 24–48 h. These values were logarithmically transformed and expressed as log_10_ CFU/mL

### 2.4. Impact of Treatment with Immobilised Antimicrobials on Apple Juice Safety and Quality

#### 2.4.1. Apple Juice Preparation

Apple juice was prepared using ‘Fuji’ apples (*Malus domestica*). The apples, purchased from a local supermarket, were processed using a Thermomix TM31 (Vormerk MSL, Madrid, Spain). After peeling and chopping, the apples were blended with sterile distilled water at a ratio of 1 L of water per kilogram of apples. The resulting mixture was then passed through a 100 µm stainless-steel strainer without applying pressure to remove the fruit pulp and produce fresh juice. The juice was portioned into aliquots and frozen at −40 °C for storage.

#### 2.4.2. Determination of the Antibacterial Activity of Immobilised Antimicrobials in Juice

The antimicrobial activity of the immobilised EOCs when incorporated into fresh apple juice was assessed following the methodology described in Section 2.3.2.

#### 2.4.3. Modelling the Inactivation Kinetics

After determining the antibacterial activity of the functionalised particles, survival curves were fitted to the Weibull model using Statgraphics Centurion XIX (Statpoint Technologies, Inc., Warrenton, VA, USA):

(1) $$logS=-{\left(\frac{t}{a}\right)}^{n}$$

where *S* is the survival fraction (N/N_0_), and *a* and *n* are the scale and shape factors, respectively; the *n* factor describes the shapes of the survival curve insofar as when *n* < 1, the survival curve is concave upwards, *n* > 1 denotes that the survival curve is concave downwards, and *n* = 1 means a straight line on a logarithmic scale.

The model was estimated using nonlinear least squares by assuming that residuals were randomly distributed following normal distribution with an average equalling zero [29].

The criteria used to evaluate the models’ goodness of fit were adjusted R^2^ and mean square error (MSE). Corrected R^2^ and MSE were calculated to check how well the models fit the experimental data. The accuracy factor (*A_f_*) parameter was obtained to externally validate the models with a replicate not used to build them. This factor provides an idea of the model’s accuracy to predict experimental data, and can be defined as follows [30]:

(2) Af=10∑logpredictedobservedn

where *n* is the number of observations, and the predicted and observed values refer to the survival fraction. The meaning of this statistic is the closer to 1 the *A_f_* values, the better the model fits the data.

#### 2.4.4. Sensory Evaluation

Triangle tests were conducted to assess the panellists’ ability to distinguish between fresh juice and the treated apple juices containing the free and immobilised EOCs. In these triangular presentations, panellists received three coded samples: two samples of the same type and one different sample. Samples were presented in randomised order to ensure that the selection of the odd sample occurred with a probability of one third. Each juice sample, measuring 10 mL, was served at room temperature in a capped transparent glass vial labelled with a random three-digit code to evaluate general appearance, while amber vials were used to assess aroma.

### 2.5. Statistical Analysis

Data were statistically processed by Statgraphics Centurion XIX (Statpoint Technologies, Inc., Warrenton, VA, USA). The influence of the different EOCs and the concentrations of the immobilised EOCs on bacterial viability were analysed by a multifactor analysis of variance (ANOVA). The LSD (least significant difference) procedure was followed to test the differences between averages at the 5% significance level.

## 3. Results and Discussion

### 3.1. The MIC and the MBC of the Free EOCs

The MIC and the MBC of the free EOCs against foodborne pathogens (*E. coli*, *S. enterica*, *S. aureus* and *L. monocytogenes*) are shown in Table 1. All of the EOCs exhibited antibacterial activity against the tested microorganisms, but at different degrees. Carvacrol and thymol had similar MIC and MBC values against bacteria, and exhibited a wide range of antibacterial activity. These were very effective against *S. enterica* and had the lowest MIC and MBC values (0.3125 and 0.625 mg/mL, respectively). In contrast, their effectiveness against *L. monocytogenes* was less (MIC and MBC of 1.25 and 2.5 mg/mL, respectively). The mildest antimicrobial effect was for eugenol, with MIC values ranging from 2.5 to 5 mg/mL and MBC values from 5 to 10 mg/mL, depending on the target microorganism. *L. monocytogenes* was the most resistant pathogen.

These findings are consistent with those of Pei [31], who reported that carvacrol was more effective against *E. coli* than eugenol. Similarly, Liu [32] observed that thymol and carvacrol were more effective than eugenol against *S. typhimurium*. Gallucci [33] attributed the differences in antimicrobial activity between carvacrol, thymol (both phenolic terpenoids), and eugenol (a phenylpropane derivative) to variations in hydrogen-bonding capacity. Eugenol can form intramolecular hydrogen bonds, which reduces its ability to form intermolecular bonds, and thereby diminishes its antimicrobial efficacy.

Finally, the greater resistance of *L. monocytogenes* to the free EOCs observed in our study is corroborated by other studies. Liu [32] observed that *L. monocytogenes* was more resistant to thymol than *S. typhimurium*. Gan [34] noted that *S. aureus* was more resistant to thymol than *S. typhimurium*, and more sensitive than *L. monocytogenes*.

Due to the poorer antimicrobial activity of eugenol against the tested foodborne pathogens, only thymol and carvacrol were immobilised on the surface of the SiO_2_ microparticles (5–15 µm) by the Mannich reaction.

### 3.2. Thymol and Carvacrol Immobilisation

#### 3.2.1. Optimisation of the Synthesis Conditions

The Mannich reaction requires a compound with an amine group (primary or secondary) to react with the nucleophile (thymol or carvacrol) in the presence of formaldehyde to form a Mannich base. In this case, APTES, a molecule containing a secondary amine group, was used for the silanisation of the surfaces. When the SiO_2_ particles functionalised with APTES were exposed to thymol or carvacrol in the presence of formaldehyde, this facilitated the anchoring of the antimicrobial compound onto the particle. However, this compound, in excess, should be efficiently eliminated by washing. Therefore, before studying the effect of the EOCs immobilised on silica particles by the Mannich reaction, we investigated the minimal formaldehyde concentration needed to derivatise the antimicrobial compound and to allow for its anchorage onto the material’s surface. In this case, we selected thymol as a natural antimicrobial component. Bearing this in mind, the number of washes until there was no leaching EOCs and formaldehyde, the amount of EOCs anchored onto the silica surface and the antimicrobial activity of the proposed materials against *E. coli* in PBS were evaluated.

Table 2 shows the number of washes and the degree of functionalisation of particles needed until no formaldehyde and thymol leaching was detected. It revealed that the higher the formaldehyde concentration, the more washes needed to remove this molecule from media. The degree of functionalisation was significantly lower in the reaction with the smallest amount of formaldehyde (reaction E) (*p* < 0.05), probably due to insufficient formaldehyde to modify and anchor thymol onto the surface. At higher formaldehyde concentrations (reactions A to D), the degree of functionalisation remained consistent, and no significant differences were observed (*p* > 0.05).

**Figure 2:**
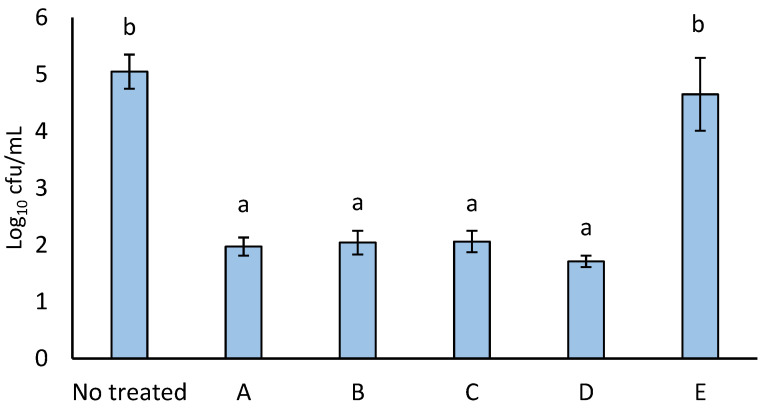
Effect of the different reactions of thymol immobilisation on *E. coli* growth. Mean value ± SD (n = 3). Different small letters indicate significant differences in the *E. coli* counts among the immobilisation reactions (*p* < 0.05).

Following the evaluation of how the reagent concentration affected the degree of particle functionalisation, the antimicrobial activity of the immobilised thymol was assessed. As illustrated in Figure 2, the treatment with immobilised thymol resulted in approximately a 3.5 log_10_ reduction in bacterial growth for reactions A to D (*p* > 0.05). In this case, the same antimicrobial results for these reactions were consistent with there being no significant changes in the degree of functionalisation. With reaction E, no antimicrobial activity was observed, possibly due to the lower degree of functionalisation, which may prevent contact between the EOC and the microorganism.

Bearing in mind all of these results, reaction D was identified as the optimal candidate for in vitro studies as it required fewer washes and there was a similar degree of functionalisation and antimicrobial activity to other reactions.

#### 3.2.2. Preparation and Characterisation of the Antimicrobial Particles

**Figure 3:**
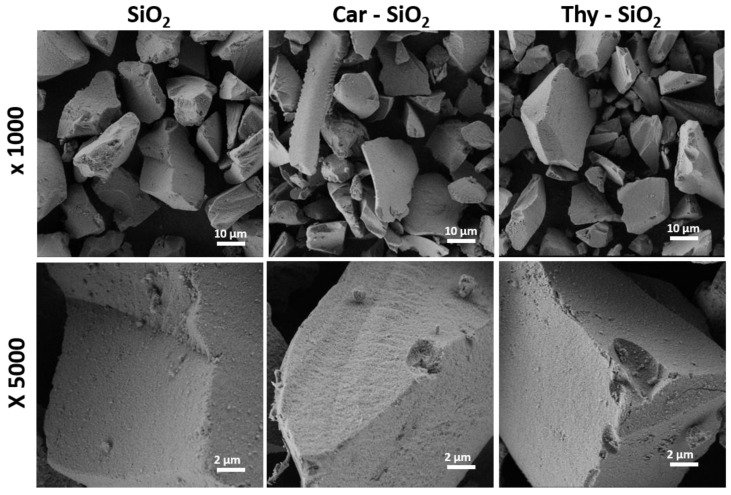
Characterisation of particle size and particle shape by FESEM of the bare and EOC-functionalised mesoporous silica particles.

After determining the optimised synthesis conditions, the SiO_2_ particles were functionalised with thymol and carvacrol to yield the corresponding **SiO_2_-Thy** and **SiO_2_-Car** particles. Then, the particles were characterised by standard techniques. The morphological characterisation of the bare and EOC-functionalised silica particles by FESEM is shown in Figure 3. No changes in the size, shape, and surface of the supports were detected when comparing the bare and functionalised particles, which confirmed that the immobilisation process did not affect the integrity of the supports. Similarly, other covalent immobilisation strategies on silica supports have shown no differences in the morphology of the functionalised particles [12,13].

**Figure 4:**
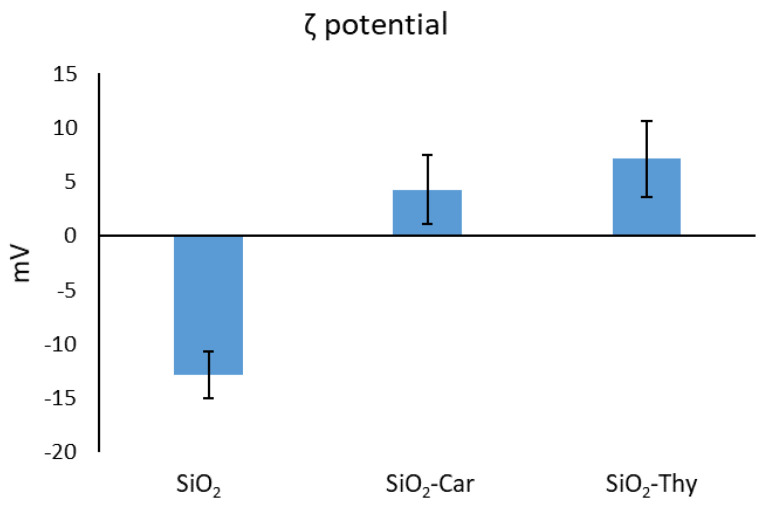
Zeta potential of the bare and EOC-functionalised mesoporous silica particles.

Additionally, as shown in Figure 4, the bare and functionalised mesoporous silica particles presented ζ values ranging from −30 mV to +30 mV. After functionalisation, the ζ values changed from negative to positive values, which corroborated the attachment of the different EOCs to the mesoporous particles [12]. The degree of functionalisation was approximately 45.7 ± 4.7 mg/g SiO_2_ for Car and 55.3 ± 5.2 mg/g SiO_2_ for Thy. These values surpass those previously reported in other studies that have applied conventional functionalisation based on paraformaldehyde formylation [14]. These values were used to calculate the quantities of the **SiO_2_-EOCs** equivalent to the MIC of the free Car or Thy.

### 3.3. Antibacterial Activity of the Immobilised Antimicrobials in PBS

Having synthesised and characterised the **SiO_2_-Thy** and **SiO_2_-Car** particles obtained by the Mannich reaction, their antimicrobial potential against the four bacterial strains under study was evaluated. In this section, PBS was used as the culture medium.

**Figure 5:**
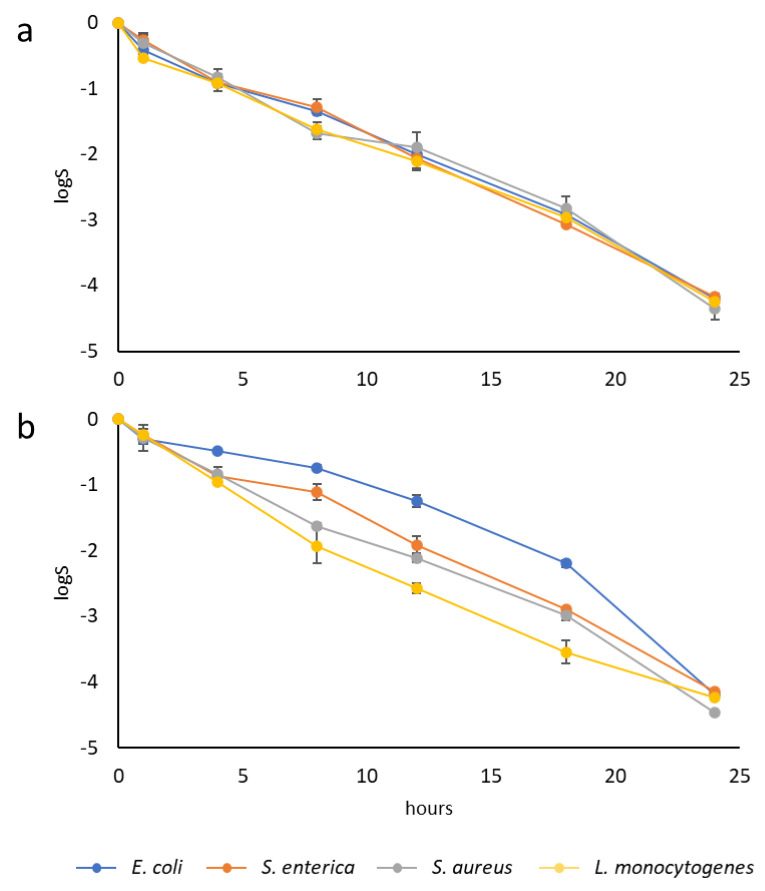
Inactivation of foodborne pathogens in PBS treated with immobilised EOCs (thymol (**a**) and carvacrol (**b**)) at the MBC.

Figure 5 depicts the inactivation curves for the foodborne pathogens during their incubation at 37 °C with the immobilised thymol or carvacrol at an equivalent concentration to the MBC. As illustrated, both the immobilised EOCs exhibited antimicrobial activity against all of the pathogens. On all curves, inactivation increased with a longer incubation time, and the reduction was more than 4 log cycles after 24 h.

The treatments with **SiO_2_-Thy** yielded similar reductions in microbial counts across all of the tested microorganisms (*p* > 0.05). In contrast, the effectiveness of the Car-functionalised particles varied, depending on the evaluated microorganism. The **SiO_2_-Car** particles were more effective against L. monocytogenes in short incubation times (t < 24 h).

In line with this, the covalent immobilisation of thymol and carvacrol on silica microparticles by the Mannich reaction has proven antimicrobial effectiveness that is comparable to the effectiveness demonstrated by EOCs immobilised by other reactions in previous works. Ribes [13] found that MCM-41 microparticles functionalised with carvacrol and eugenol inhibited *E. coli* growth in apple and grape juice. Liu [14] demonstrated the antimicrobial activity of thymol immobilised on hollow mesoporous silica particles against *E. coli* in apple juice. The use of EOCs immobilised on silica microparticles as food processing aids has also been proven to be effective in eliminating *E. coli* in apple juice [12] and spoilage microorganisms in white wine [17]. Despite being isomers, the difference in inactivation achieved between thymol and the immobilised carvacrol may be because the hydroxyl group of carvacrol is less accessible than the hydroxyl group of thymol. Ribes [35] observed that particles functionalised with thymol or carvacrol had different antimicrobial activities against *E. coli*, *H. pylori*, *L. pneumophila*, and *P. aeruginosa* in water.

### 3.4. Antibacterial Activity of Immobilised Antimicrobials in Fresh Apple Juice

The previous section indicated the finding that the synthesised particles had high antimicrobial capacity against the four strains in PBS. However, it is well-known that the antimicrobial efficacy of free EOCs when incorporated into food depends on the physico-chemical characteristics (pH, temperature,) and composition of food (proteins, fats, carbohydrates, etc.) [36]. Thus to check the effect of apple juice composition on the effectiveness of the particles, the **SiO_2_-Thy** and **SiO_2_-Car** particles were allowed to come into contact with the pathogenic microorganisms in fresh apple juice.

**Figure 6:**
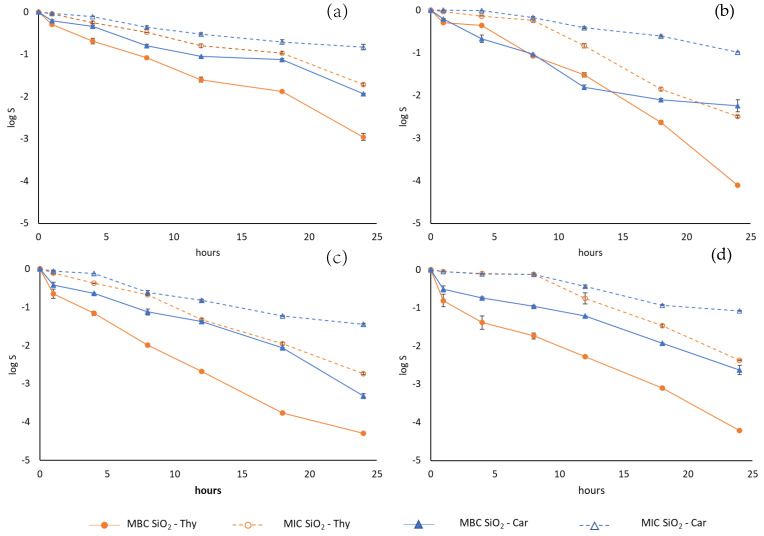
Survival curves of foodborne pathogens (*E. coli* (**a**); *S. enterica* (**b**); *S. aureus* (**c**); *L. monocytogenes* (**d**)) in fresh apple juice treated with the immobilised EOCs. MIC and MBC of SiO_2_-Thy and SiO_2_-Car correspond to the particle concentrations equivalent to the minimum inhibitory and bactericidal concentrations of thymol and carvacrol.

According to Figure 6, these two particles exhibited remarkable antimicrobial activity against the four pathogenic strains of interest, which demonstrates the effectiveness of the reaction. However, the particles’ antimicrobial activity varied based on the type of immobilised EOC and the target microorganism. In all cases, the particles exhibited less antimicrobial effectiveness in juice compared to PBS. The **SiO_2_-Car** particles were generally more adversely affected than **SiO_2_-Thy**. Furthermore, for the Thy particles, less antimicrobial activity was observed against *E. coli* (*p* < 0.05) and *S. enterica* (*p* < 0.05). Gómez-Llorente [37] found that the presence of certain constituents (proteins, lipids, or carbohydrates) of foods produced a loss of antimicrobial capacity for the particles functionalised with vanillin against *E. coli* K12. With natural apple juice, the large amount of pectin present in juice may be responsible for the decreased antimicrobial activity of the **SiO_2_-Thy** particles against *E. coli* and *S. enterica*. The increased activity of the thymol-functionalised particles against *L. monocytogenes* and *S. aureus* may be due to these microorganisms’ sensitivity to the acidic pH of apple juice, despite the blocking effect of pectin. Differences between Car and Thy might be due to the different anchoring conformation of carvacrol, which likely increases the probability of its hydroxyl group being obstructed by food components.

The phenotypic heterogeneity of microbial populations can result in nonlinear inactivation, which complicates the calculation of microbial death [38]. Given the shape of the inactivation curves, the antimicrobial effect of the immobilised EOCs was further evaluated by fitting the experimental results to the Weibull distribution function.

Table 3 shows the a (scale factor) and n (shape factor) values, and the R^2^, MSE, and A_f_ values when fitting the survival curves to the Weibull distribution function. The n values, which are both higher and less than 1, indicate that survival patterns are either concave or convex depending on the treatment conditions and pathogen. However, as the immobilised thymol concentration rises, the n value lowers. The value of a, which is inversely related to the inactivation rate, was observed for all pathogens, and increasing the concentration of the immobilised EOCs made the microbial inactivation rate significantly higher (*p* < 0.05). Except for *S. enterica*, the inactivation rate was lower (*p* < 0.05) when using thymol compared to the immobilised carvacrol. Therefore, these pathogens exhibit greater resistance to carvacrol.

The criteria used to evaluate the models’ goodness of fit were adjusted R^2^ and MSE. Corrected R^2^ and MSE were calculated to check how well the models fit the experimental data. The Af parameter was obtained to externally validate the models with a replicate not used to build them. This factor provides an idea of the model’s accuracy to predict the experimental data, and can be defined as follows [30]:

The model’s suitability for fitting the experimental data was evaluated using R^2^ and MSE (Table 3). The fitted values closely matched the experimental ones, with an adjusted R^2^ ranging from 0.996 to 0.958. The obtained regression coefficients were significant at the 95% confidence level in all cases. According to these results, the Weibull model demonstrated an acceptable goodness-of-fit for each studied parameter and pathogen.

To assess the model’s predictive capability for forecasting microbial responses, the A_f_ was used [30,39]. Table 3 presents the A_f_ values for each combination of EOC, EOC concentration, and pathogen. The A_f_ values ranged from 1.047 to 1.117, which indicate a high precision level for predictions, with an error margin of 4.7–11.7%.

Kinetic parameters and models are used to undertake food preservation processes to ensure safety. They also provide tools to compare the impact of the different process parameters of preservation technologies on reducing microbial populations. The present study is the first to provide a mathematical model of the survival of foodborne pathogens when exposed to EOCs immobilised on silica particles. The survival curves in this study displayed nonlinear behaviour, and the presence of shoulders on curves was noted under certain conditions. This type of behaviour is common in non-heat preservation processes and requires using mathematical models other than the log-linear model [40]. The Weibull distribution function was applied for its simplicity and robustness to describe the inactivation kinetics [41]. The Weibull model presented a good fit to the survival curves of the studied pathogens, which highlights that the concentration of immobilised EOC affected the inactivation rate of all of the studied pathogens.

### 3.5. Impact of Incorporating Immobilised EOCs into the Sensory Analysis of Apple Juice

Finally, the sensory evaluation of the immobilised EOCs in fresh apple juice was conducted. In visual assessment terms, it was evident that the addition of free EOCs did not produce any noticeable changes in our samples. In contrast, the inclusion of the immobilised EOCs allowed consumers to differentiate between the treated and fresh samples. However, they were unable to identify which samples were treated and which were not because both types exhibited typical fresh apple juice characteristics despite slight differences. Regarding aroma, 100% of the tasters recognised differences between the fresh juices and those containing free EOCs such as Car or Thy. Conversely, they did not perceive these differences when the EOCs were immobilised.

From this, it can be concluded that the addition of free EOCs negatively impacts the juice aroma and acceptance. Conversely, although employing the immobilised EOCs in natural apple juice effectively inactivated pathogens, it did not demonstrate any effect on the evaluated sensory attributes. This outcome aligns with the optimisation process of the immobilisation reaction because one of the goals was to prevent EOC migration into juice. Other authors have observed that the addition of EOC immobilised on silica supports did not alter the organoleptic properties of fruit juices [11,12]. Therefore, utilising EOCs immobilised on silica microparticles as a food preservative maintains the antimicrobial capacity of EOCs without modifying the organoleptic properties of natural apple juice.

## 4. Conclusions

In this study, the Mannich reaction proved to be a valid strategy for immobilising thymol and carvacrol on silica microparticles. The use of the Mannich reaction simplified the immobilisation process and reduced the number of reagents compared to other covalent immobilisation strategies. The immobilisation of thymol and carvacrol on silica particles by the Mannich reaction is an excellent proven strategy to create effective food preservatives capable of improving the microbiological food safety of apple juice without altering the nutritional sensory properties.

The antimicrobial systems developed, consisting of food-grade molecules like thymol or carvacrol combined with biocompatible silica microparticles, offer versatile applications in the food industry. These may function as food preservatives integrated into products or as processing aids, where the silica supports are removed after completing microbial stabilization.

However, before real-world applications, further research is needed to evaluate their efficacy against other spoilage and pathogenic microorganisms in apple juice as well as to assess the impact of the treatment on the juice properties during processing and storage.

## Figures and Tables

**Table 1:** The MIC and MBC values (expressed as mg/mL) against foodborne pathogens.

	Eugenol		Carvacrol		Thymol	
	MIC	MBC	MIC	MBC	MIC	MBC
*E. coli*	2.5	7.5	0.625	1.25	0.625	1.25
*S. enterica*	2.5	7.5	0.3125	0.625	0.3125	0.625
*S. aureus*	5	5	0.625	1.25	0.625	1.25
*L. monocytogenes*	2.5	10	1.25	2.5	1.25	2.5

**Table 2:** Number of washes and degree of functionalisation (mg EOCs/g SiO_2_) after no leaching of EOCs and formaldehyde. Mean values ± SD (n = 3).

Reaction	Molar Relation (thy:formaldehyde:SiO_2_-APTES)	Number of Washes	Degree of Functionalisation (mg Thy/g SiO_2_)
A	(1:1:1)	103 ± 13 ^e^	48.27 ± 2.12 ^b^
B	(1:0.75:1)	81 ± 10 ^d^	48.73 ± 3.75 ^b^
C	(1:0.50:1)	61 ± 7 ^c^	49.68 ± 5.68 ^b^
D	(1:0.25:1)	34 ± 3 ^b^	55.34 ± 5.20 ^b^
E	(1:0.10:1)	10 ± 2 ^a^	26.42 ± 1.94 ^a^

Different small letters in the same column indicate significant differences in the number of washes or degree of functionalisation between reactions (*p* < 0.05).

**Table 3:** Parameters ± standard error, MSE, and Af of the Weibull model for the foodborne pathogens’ survival curves. Different small letters indicate significant differences in the counts among pathogens (*p* < 0.05). Different capital letters denote significant differences in the counts among EOC concentrations (*p* < 0.05). Different numbers refer to significant differences in the counts among EOCs (*p* < 0.05).

Pathogen	EOC	[EOC] mg/mL	a	n	R^2^	MSE	A_f_
*E. coli*	Thy	MIC	15.81 ± 0.14 ^aA1^	1.16 ± 0.01 ^aA1^	97.48	0.011	1.100
		MBC	7.18 ± 0.12 ^aB1^	0.85 ± 0.04 ^aB1^	97.64	0.035	1.072
	Car	MIC	28.13 ± 0.95 ^aA2^	0.88 ± 0.07 ^aA2^	97.64	0.007	1.082
		MBC	12.13 ± 0.18 ^aB2^	0.84 ± 0.04 ^aA1^	95.50	0.026	1.109
*S. enterica*	Thy	MIC	13.32 ± 0.08 ^bA1^	1.62 ± 0.01 ^bA1^	98.29	0.026	1.066
		MBC	8.43 ± 0.13 ^bB1^	1.33 ± 0.01 ^bB1^	99.24	0.026	1.049
	Car	MIC	24.34 ± 0.02 ^bA2^	1.50 ± 0.09 ^bA2^	99.03	0.002	1.117
		MBC	6.39 ± 0.15 ^bB2^	0.62 ± 0.06 ^bB2^	96.14	0.042	1.077
*S. aureus*	Thy	MIC	9.97 ± 0.25 ^cA1^	1.15 ± 0.02 ^aA1^	99.66	0.006	1.048
		MBC	2.81 ± 0.20 ^cB1^	0.55 ± 0.17 ^cB1^	99.14	0.066	1.049
	Car	MIC	15.42 ± 0.35 ^cA2^	0.94 ± 0.02 ^aA2^	97.84	0.010	1.049
		MBC	8.75 ± 1.36 ^cB2^	1.11 ± 0.13 ^cB2^	97.26	0.074	1.047
*L. monocytogenes*	Thy	MIC	14.96 ± 0.54 ^aA1^	1.86 ± 0.13 ^cA1^	98.88	0.022	1.088
		MBC	3.02 ± 0.69 ^cB1^	0.66 ± 0.08 ^acB1^	97.39	0.060	1.063
	Car	MIC	21.65 ± 0.23 ^dA2^	1.38 ± 0.03 ^bA2^	95.78	0.015	1.065
		MBC	7.78 ± 0.10 ^cbB2^	0.80 ± 0.03 ^abB2^	95.96	0.036	1.089

EOC: essential oil component; [EOC]: EOC concentration; a: scale factor; n: shape factor; R^2^: coefficient of determination. MSE: mean square error; Af: accuracy factor. Thy: thymol; Car: carvacrol; MIC: minimum inhibitory concentration; MBC: minimum bactericidal concentration.

## Data Availability

The original contributions presented in the study are included in the article, further inquiries can be directed to the corresponding author.
